# Emerging genotype–phenotype relationships in patients with large *NF1* deletions

**DOI:** 10.1007/s00439-017-1766-y

**Published:** 2017-02-17

**Authors:** Hildegard Kehrer-Sawatzki, Victor-Felix Mautner, David N. Cooper

**Affiliations:** 1 Institute of Human Genetics, University of Ulm, Albert-Einstein-Allee 11, 89081 Ulm, Germany; 2 Department of Neurology, University Hospital Hamburg Eppendorf, 20246 Hamburg, Germany; 3 Institute of Medical Genetics, School of Medicine, Cardiff University, Cardiff, CF14 4XN UK

## Abstract

The most frequent recurring mutations in neurofibromatosis type 1 (NF1) are large deletions encompassing the *NF1* gene and its flanking regions (*NF1* microdeletions). The majority of these deletions encompass 1.4-Mb and are associated with the loss of 14 protein-coding genes and four microRNA genes. Patients with germline type-1 *NF1* microdeletions frequently exhibit dysmorphic facial features, overgrowth/tall-for-age stature, significant delay in cognitive development, large hands and feet, hyperflexibility of joints and muscular hypotonia. Such patients also display significantly more cardiovascular anomalies as compared with patients without large deletions and often exhibit increased numbers of subcutaneous, plexiform and spinal neurofibromas as compared with the general NF1 population. Further, an extremely high burden of internal neurofibromas, characterised by >3000 ml tumour volume, is encountered significantly, more frequently, in non-mosaic *NF1* microdeletion patients than in NF1 patients lacking such deletions. *NF1* microdeletion patients also have an increased risk of malignant peripheral nerve sheath tumours (MPNSTs); their lifetime MPNST risk is 16–26%, rather higher than that of NF1 patients with intragenic *NF1* mutations (8–13%). *NF1* microdeletion patients, therefore, represent a high-risk group for the development of MPNSTs, tumours which are very aggressive and difficult to treat. Co-deletion of the *SUZ12* gene in addition to *NF1* further increases the MPNST risk in *NF1* microdeletion patients. Here, we summarise current knowledge about genotype–phenotype relationships in *NF1* microdeletion patients and discuss the potential role of the genes located within the *NF1* microdeletion interval whose haploinsufficiency may contribute to the more severe clinical phenotype.

## Introduction

Neurofibromatosis type 1 (NF1; MIM#162200) is a tumour predisposition syndrome with an incidence at birth of 1 in 2000–3000 (Crowe et al. 1956; Lammert et al. 2005; Uusitalo et al. 2015). The hallmark features of NF1 are café-au-lait spots (CALS) and the pathognomonic neurofibromas. The majority of NF1 patients are characterised by mutations residing within the boundaries of the *NF1* gene, which spans 287-kilobases (kb) of chromosome 17q11.2 and comprises 57 constitutive and 3 alternatively spliced exons.

Only a few genotype–phenotype correlations in NF1 have been identified to date. One of these relates to spinal neurofibromatosis (SNF) which is characterised by bilateral neurofibromas located at all 38 spinal nerve roots. The risk of having SNF versus NF1 without spinal neurofibromas, or NF1 with neurofibromas affecting only some but not all spinal nerve roots, is significantly increased in individuals harbouring *NF1* missense mutations (Ruggieri et al. 2015). Furthermore, the recurrent three base-pair in-frame deletion, c.2970-2972 delAAT, within exon 17 of the *NF1* gene leads to the loss of a single amino acid (p.Met992del) and is associated with a relatively mild NF1 phenotype that is characterised by the occurrence of CALS and skinfold freckling but a lack of externally visible cutaneous or plexiform neurofibromas (Upadhyaya et al. 2007). The second well-established genotype–phenotype correlation in NF1 is associated with missense mutations affecting codon p.Arg1809. Individuals with these very specific missense *NF1* mutations exhibit CALS (with or without freckling) and Lisch nodules, but no externally visible plexiform neurofibromas or cutaneous neurofibromas (Pinna et al. 2015; Rojnueangnit et al. 2015). Approximately, 25% of the individuals with missense mutations affecting codon p.Arg1809 have Noonan-like features including pulmonic stenosis and short stature whilst 50% of them exhibit developmental delay and/or learning disability (Rojnueangnit et al. 2015). However, missense mutations affecting codon p.Arg1809 appear to be quite rare, since they were observed in only 1.2% of the cohort of 7000 NF1 patients with identified mutations. In the same cohort of patients, the prevalence of the recurrent one amino acid deletion (p.Met992del) was 0.8% (Rojnueangnit et al. 2015).

The third genotype–phenotype relationship evident in NF1 is that associated with large *NF1* deletions and is the topic of this review. An estimated 4.7–11% of all NF1 patients have large deletions encompassing the entire *NF1* gene and its flanking regions at 17q11.2 (Cnossen et al. 1997; Rasmussen et al. 1998; Kluwe et al. 2004; Zhang et al. 2015). Large deletions of the *NF1* gene and its flanking regions (generally termed ‘*NF1* microdeletions’) are frequently associated with a severe clinical manifestation of NF1 as described below.

**Fig. 1:**
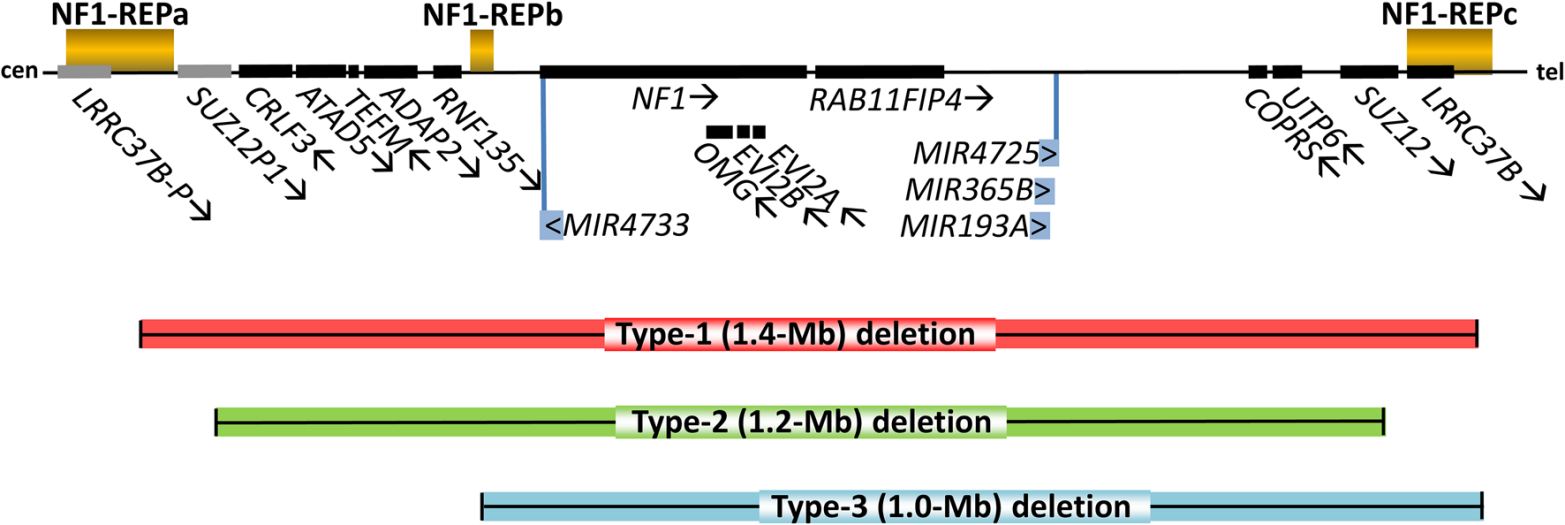
Schema of the genomic region at 17q11.2 harbouring the *NF1* gene and its flanking genes included within the boundary of the type-1 *NF1* deletion interval encompassing 1.4-Mb (*red bar*). The *arrows* given subsequent to the symbols of the genes denote their transcriptional orientation. *SUZ12P1* and *LRRC37B*-*P* are non-functional pseudogenes. *cen* centromeric direction, *tel* telomeric direction

Altogether, four types of large *NF1* deletion (type-1, 2, 3 and atypical) have been identified that are distinguishable in terms of their size and breakpoint location, by the number of genes located within the deletion region or by the frequency of somatic mosaicism with normal cells not harbouring the deletion. Most frequent are the type-1 *NF1* deletions which encompass 1.4-Mb and include 14 protein-coding genes as well as four microRNA genes (Fig. 1) (Dorschner et al. 2000; Jenne et al. 2001; López-Correa et al. 2001). Type-1 deletions account for 70–80% of all large *NF1* deletions and usually occur as germline deletions that are present in all cells of the affected patients (Messiaen et al. 2011). Most type-1 *NF1* deletions are caused by interchromosomal non-allelic homologous recombination (NAHR) during maternal meiosis (López-Correa et al. 2000; Steinmann et al. 2008). The NAHR events causing type-1 *NF1* deletions are mediated by the low-copy repeats, NF1-REPa and NF1-REPc. Within these low-copy repeats, recurrent breakpoints have been detected within two NAHR hotspots, termed paralogous recombination sites 1 and 2 (Forbes et al. 2004; De Raedt et al. 2006; Bengesser et al. 2014; Hillmer et al. 2016).

In contrast to type-1 *NF1* deletions, type-2 deletions encompass only 1.2-Mb and are associated with hemizygosity for 13 protein-coding genes since the *LRRC37B* gene is absent from the deleted region (Fig. 1). At least 10% of large *NF1* deletions are type-2 but this is very likely to be an underestimate (Messiaen et al. 2011). Type-2 deletions are also mediated by NAHR but in contrast to type-1 *NF1* deletions, their breakpoints are located within *SUZ12* and its highly homologous pseudogene *SUZ12P1* which flank NF1-REPc and NF1-REPa, respectively (Fig. 1) (Petek et al. 2003; Vogt et al. 2012). Type-2 *NF1* deletions are frequently of postzygotic origin, mediated by mitotic NAHR, and hence are associated with somatic mosaicism of normal cells lacking the deletion (Kehrer-Sawatzki et al. 2004; Steinmann et al. 2007; Roehl et al. 2010, 2012).

Type-3 *NF1* deletions are very rare; these 1.0-Mb deletions occur in only 1-4% of all patients with gross *NF1* deletions and are mediated by NAHR between NF1-REPb and NF1-REPc leading to hemizygosity for a total of nine protein-coding genes (Fig. 1) (Bengesser et al. 2010; Pasmant et al. 2010; Zickler et al. 2012). As their name suggests, atypical large *NF1* deletions do not exhibit recurrent breakpoints and are quite heterogeneous in terms of their size and the number of genes located within the deleted region (Upadhyaya et al. 1996; Cnossen et al. 1997; Dorschner et al. 2000; Kehrer-Sawatzki et al. 2003, 2005, 2008; Venturin et al. 2004a; Gervasini et al. 2005; Mantripragada et al. 2006; Pasmant et al. 2008, 2009, 2010; Vogt et al. 2014). It has been estimated that 8–10% of all large *NF1* deletions are atypical (Pasmant et al. 2010; Messiaen et al. 2011). Atypical *NF1* deletions may occur as germline mutations but can also be of postzygotic origin and hence may be associated with somatic mosaicism with normal cells (Taylor Tavares et al. 2013; Vogt et al. 2014). Atypical *NF1* deletions are not only highly heterogeneous in terms of their length but also in terms of their underlying mutational mechanisms which may involve aberrant DNA double strand break repair and/or replication and retrotransposon-mediated mechanisms (Vogt et al. 2014 and references therein). The architecture of the genomic regions flanking the *NF1* gene in 17q11.2, characterised by low-copy repeats, predisposes to large deletions mediated by various different mutational mechanisms occurring in the germline of an unaffected parent or during mitotic postzygotic cell divisions.

*NF1* microdeletions are important from the clinical standpoint because, as noted above, they are often associated with more severe manifestations of NF1 than those noted in patients with intragenic *NF1* mutations. One interpretation of this observation is that some of those genes co-deleted with *NF1* exert an influence on the clinical manifestation of the disease in patients with *NF1* microdeletions. In the following, we shall review current knowledge about *NF1* microdeletions in terms of potential genotype–phenotype relationships and the putative modifier role of genes located within the *NF1* microdeletion interval.

## Genotype–phenotype relationships in patients with *NF1* microdeletions

The *NF1* gene was identified more than 25 years ago (Viskochil et al. 1990; Wallace et al. 1990). Genotype–phenotype analyses suggested from very early on that patients with *NF1* microdeletions often exhibit a more severe clinical phenotype than patients with intragenic *NF1* mutations; the former are frequently characterised by dysmorphic facial features and severe developmental delay (Kayes et al. 1992, 1994; Wu et al. 1995, 1997, 1999; Riva et al. 1996, 2000; Upadhyaya et al. 1996, 1998; Leppig et al. 1997; Tonsgard et al. 1997; Valero et al. 1997; Rasmussen et al. 1998; Streubel et al. 1999; Dorschner et al. 2000; Kobayashi et al. 2012).

Subsequent follow-up studies confirmed that these deletions frequently lead to severe clinical manifestations of the disease including a high tumour load and cardiovascular anomalies (Venturin et al. 2004b; Mensink et al. 2006; Pasmant et al. 2010; Zhang et al. 2015). Although, as a group, *NF1* microdeletion patients tend to exhibit a comparatively severe form of NF1, some variability of clinical symptoms has nevertheless been observed when comparing individuals with *NF1* microdeletions. These clinical phenotypic differences may be influenced by the variable expressivity of NF1 characteristic of all NF1 patients, irrespective of the type of *NF1* gene mutation involved (Sabbagh et al. 2009; reviewed by Pasmant et al. 2012). However, a certain proportion of the phenotypic variability exhibited by patients with *NF1* microdeletions who were investigated in the above-mentioned studies may have been due to differences in deletion size and breakpoint location which together determine the number of genes included within the deletion interval.

Somatic mosaicism with cells not harbouring the *NF1* microdeletion in question is likely to have a disproportionately large impact upon the manifestations of disease (Rasmussen et al. 1998; Tinschert et al. 2000; Maertens et al. 2007; Kehrer-Sawatzki and Cooper 2008; Roehl et al. 2012; Kehrer-Sawatzki et al. 2012). This is particularly relevant since some types of *NF1* microdeletion, such as the type-2 and atypical *NF1* deletions, are frequently of postzygotic origin and hence occur as mosaic deletions alongside normal cells in the body of an affected patient (Steinmann et al. 2007; Vogt et al. 2014). By contrast, type-1 *NF1* deletions are only very rarely of postzygotic origin (Messiaen et al. 2011). This notwithstanding, in many of the reported studies of genotype–phenotype correlations in patients with *NF1* microdeletions, neither deletion size nor somatic mosaicism has been specifically taken into consideration.

To refine the genotype–phenotype analysis of *NF1* microdeletions, Mautner et al. (2010) investigated 29 NF1 patients with non-mosaic type-1 (1.4-Mb) *NF1* deletions. A combination of breakpoint-spanning PCRs and/or polymorphic marker analysis confirmed that the deletion breakpoints were located within specific regions of the NF1-REPs. Thus, all 29 patients studied were hemizygous for the same number of genes at 17q11.2 (Fig. 1). The clinical analysis of these 29 patients with type-1 *NF1* microdeletions served to confirm that several clinical phenotypic features were relatively frequent in patients with *NF1* microdeletions but less common (or even not identifiable) in the general NF1 population (as may be concluded from the studies listed in Table 1). These features included facial dysmorphism, overgrowth/tall-for-age stature, significant delay in cognitive development, scoliosis, bone cysts, large hands and feet with excessive soft tissue, hyperflexibility of joints of hands and feet and pronounced muscular hypotonia. Further, patients with type-1 *NF1* microdeletions exhibited a variety of features that were markedly more frequent than in the general NF1 population including intellectual disability, high numbers of subcutaneous and spinal neurofibromas, and the occurrence of plexiform neurofibromas (Table 1). An increased frequency of optic gliomas was however not observed in patients with type-1 *NF1* deletions as compared to the general NF1 population. In the studies listed in Table 1 that compared the frequency of clinical features, NF1 patients were analysed irrespective of the type of *NF1* mutation they harboured; these patients are therefore referred to as the ‘general NF1 population’. Such comparisons will tend to err on the conservative side since 4.7–11% of the individuals in the general NF1 population harbour *NF1* microdeletions.

**Table 1:** Frequency of clinical symptoms in patients with type-1 *NF1* microdeletions investigated by Mautner et al. (2010) and in the general NF1 population

Clinical features	Frequency in patients with type-1 *NF1* deletions, (%)	Frequency in the general NF1 population (reference), (%)	
Facial dysmorphism	90	n.d.	
Hypertelorism	86	n.d.	
Facial asymmetry	28	8 6	(Friedman and Birch 1997) (Sbidian et al. 2012)
Coarse face	59	n.d.	
Broad neck	31	n.d.	
Tall-for-age stature	46	n.d.	
Macrocephaly^a^	39	45 29 43 24	(Huson et al. 1988) (Riccardi 1992) (North 1993) (Sbidian et al. 2012)
Large hands and feet	46	n.d.	
Pes cavus	17	n.d.	
Café-au-lait spots	93	87 95 86 99	(McGaughran et al. 1999) (Friedman 1999) (Duong et al. 2011) (Sbidian et al. 2012)
Axillary and inguinal freckling	86	86 89	(Duong et al. 2011) (Plotkin et al. 2012)
Lisch nodules	93	63 93 50 45	(McGaughran et al. 1999) (Huson et al. 1988) (Friedman 1999) (Sbidian et al. 2012)
Significant delay in cognitive development	48	17	(Klein-Tasman et al. 2014)
General learning difficulties	45	45 47 32 39 31	(North et al. 1995) (Brewer et al. 1997)^b^ (Hyman et al. 2006) (Krab et al. 2008)^c^ (Plotkin et al. 2012)
IQ < 70	38	8 7	(Ferner et al. 1996) (Hyman et al. 2005)
Attention deficit hyperactivity disorder	33	49 38	(Mautner et al. 2002) (Hyman et al. 2005)
Skeletal anomalies	76	31	(Plotkin et al. 2012)
Scoliosis	43	26 12 10 20 25 28	(Friedman and Birch 1997) (McGaughran et al. 1999) (Riccardi 1999a) (North 2000) (Duong et al. 2011) (Plotkin et al. 2012)
Pectus excavatum	31	50 12	(Riccardi 1999b) (Castle et al. 2003)
Bone cysts	50	1	(Plotkin et al. 2012)
Hyperflexibility of joints	72	n.d.	
Excess soft tissue in hands and feet	50	n.d.	
Congenital heart defects	29	2 1.6–2	(Friedman and Birch 1997) (Lin et al. 2000)
Epilepsy	7	7 4 13 4	(Huson et al. 1988) (North 1993) (Ferner et al. 1996) (Kulkantrakorn and Geller 1998)
Muscular hypotonia	45	27	(Wessel et al. 2013)
Speech difficulties	48	25 20–55	(North 1999 n.d.) (Alivuotila et al. 2010)^d^
Subcutaneous neurofibromas	76	48	(Tucker et al. 2005)
Cutaneous neurofibromas	86	38–44 59 85 76 84	(Friedman and Birch 1997) (McGaughran et al. 1999) (Tucker et al. 2005) (Duong et al. 2011) (Plotkin et al. 2012)
Plexiform neurofibromas	76	15 44 50 30 54	(McGaughran et al. 1999) (Waggoner et al. 2000) (Ferner et al. 2007) (Duong et al. 2011) (Plotkin et al. 2012)
Malignant peripheral nerve sheath tumours	21	2–5 7	(Ferner and Gutmann 2002) (Duong et al. 2011)
Spinal neurofibromas	64	30 24	(Tucker et al. 2005) (Plotkin et al. 2012)
Optic pathway gliomas	19	15 19 14 11 18	(Lewis et al. 1984) (Listernick et al. 1997) (Duong et al. 2011) (Plotkin et al. 2012) (Millichap 2015; Prada et al. 2015)
T2 hyperintensities	45	34 77 79 71	(Ferner et al. 1993) (Itoh et al. 1994) (Sevick et al. 1992) (Hyman et al. 2007)

*n.d.* not determined; these features are either absent or rare in the general NF1 population

^a^The evaluation criteria in these studies included a definition of macrocephaly as an occipitofrontal circumference greater than the 98th centile or two standard deviations above the mean. Despite consistent evaluation criteria having been employed, a high degree of variability in terms of the frequency of macrocephaly has been observed

^b^39% of the children with NF1 analysed by Brewer et al. (1997) exhibited general learning disabilities whereas an additional 14% exhibited visuospatial-construction deficiencies (specific learning disabilities)

^c^39% of the children with NF1 investigated by Krab et al. (2008) had general learning disabilities whilst an additional 39% had specific learning disabilities

^d^ Alivuotila et al. (2010) investigated the speech characteristics of 62 NF1 patients (40 adults and 22 children) and compared them with those observed in 24 control individuals. Patients with NF1 exhibited deviations in voice quality (35% of the adult NF1 patients and 55% of the children with NF1), problems in regulating pitch (53% of the adult NF1 patients and 55% of the children), deviant nasality (20% of the adult NF1 patients and 45% of the children) and disfluency (20% of the adult NF1 patients and 41% of the children)

In addition to the previously mentioned clinical features, type-1 *NF1* microdeletion patients were found to have an increased risk of malignant peripheral nerve sheath tumours (MPNSTs) (De Raedt et al. 2003; Mautner et al. 2010) (Table 1). These findings confirmed that patients with non-mosaic type-1 *NF1* deletions exhibit, as a group, a severe form of NF1. However, even within this group of patients that are hemizygous for the same number of genes, some variability in expression of the clinical symptoms has been detected (Mautner et al. 2010). Hence, the phenotype associated with *NF1* microdeletions is likely to be influenced to a certain degree by the genetic background (e.g. the expression level of non-deleted genes), as well as by environmental factors.

It should be emphasised that many of the studies that have addressed the question of whether or not specific disease features occur disproportionately more frequently in patients with *NF1* microdeletions have employed, for the purposes of comparative analysis, frequency values for these clinical features that were derived from the general NF1 population obtained in different studies. More appropriate would have been a methodical comparative analysis of a large number of age-matched patients with and without germline type-1 *NF1* deletions investigated by standardised analytical tools. Although such comparative analyses have been attempted to assess differences in height (Ning et al. 2016), cognitive capability (Descheemaeker et al. 2004) or the frequency of cardiovascular anomalies (Nguyen et al. 2013), they have not as yet been performed methodically in the context of the number of neurofibromas and other *NF1* microdeletion-associated clinical features.

In the following, the clinical features associated with type-1 *NF1* microdeletions are explored in greater detail.

### High number of neurofibromas in patients with *NF1* microdeletions

Several studies have suggested that *NF1* microdeletion patients frequently exhibit a disproportionately high number of cutaneous and subcutaneous neurofibromas (Mensink et al. 2006 and references therein; Pasmant et al. 2010). Indeed, the 29 patients with type-1 *NF1* microdeletions investigated by Mautner et al. (2010) exhibited significantly increased numbers of subcutaneous but also spinal neurofibromas by comparison with the general NF1 population as concluded from some of the studies listed in Table 1. Additionally, externally visible plexiform neurofibromas are significantly more frequent in patients with *NF1* microdeletions than in the general NF1 population (Table 1; Mautner et al. 2010). Remarkably, 10 of 20 (50%) of the adult type-1 *NF1* microdeletion patients investigated by Mautner et al. (2010) exhibited a very high number of cutaneous neurofibromas (*N* > 1000). Such a high burden of cutaneous neurofibromas may also be present in some patients with intragenic *NF1* mutations but the precise frequency of this feature in this subgroup of NF1 patients is currently unknown. Plotkin et al. (2012) reported that 13% of 141 NF1 patients exhibited more than 500 cutaneous neurofibromas, but the precise proportions of patients harbouring either *NF1* microdeletions or intragenic *NF1* mutations among the 141 NF1 patients investigated had not been determined. A detailed comparison between both patient groups, involving the careful age-matching of patients, would be necessary to clarify whether a very high load of cutaneous neurofibromas (*N* > 1000) is encountered significantly more frequently in patients with type-1 *NF1* microdeletions as compared to patients with intragenic *NF1* mutations. In passing, it should be pointed out that counting neurofibromas one by one in NF1 patients who may have thousands of such tumours is likely to be subject to considerable intra- and inter-examiner variability, and hence the precise number of neurofibromas in each patient should always be regarded as a rough estimate. In an attempt to improve upon this state of affairs, Cunha et al. (2014) developed a new method using paper frames to quantify cutaneous neurofibromas. Combined with computerised analysis, this method could yet prove to be very useful for the comparative quantification of cutaneous neurofibromas in patients with *NF1* microdeletions and those lacking such deletions.

Several studies have suggested that patients with *NF1* microdeletions not only exhibit a high number of cutaneous neurofibromas but also an early (pre-pubertal) onset of cutaneuous neurofibroma growth (Kayes et al. 1992, 1994; Mensink et al. 2006; Leppig et al. 1997; Dorschner et al. 2000). However, a comprehensive analysis of age-matched children has not yet been performed to ascertain whether an early (pre-pubertal) onset in growth of multiple cutaneous neurofibromas is significantly more prevalent in children with *NF1* microdeletions as compared to children with intragenic *NF1* mutations.

In addition to tumours that are visible by external investigation, NF1 patients may also possess internal neurofibromas (mostly of the plexiform type) which would only be detectable by magnetic-resonance imaging (MRI). Kluwe et al. (2012) showed that an extremely high burden of internal neurofibromas, characterised by >3000 ml tumour volume as determined by whole-body MRI, was significantly more frequent in non-mosaic type-1 and type-2 *NF1* microdeletion patients than in NF1 patients with intragenic lesions (13 vs. 1%). Consequently, a readily identifiable subgroup of NF1 patients with germline *NF1* microdeletions is likely to exhibit an extremely high burden of internal tumours. These patients require special attention in terms of clinical care and surveillance since a strong association has been observed between the presence of internal neurofibromas and the occurrence of malignant peripheral nerve sheath tumours (MPNSTs) (Tucker et al. 2005; Mautner et al. 2008; Nguyen et al. 2014). MPNSTs are very aggressive, have a poor prognosis and frequently arise in pre-existing plexiform neurofibromas (Ferner and Gutmann 2002) which are often diagnosed before the age of 5, suggesting that they are congenital lesions (Waggoner et al. 2000). This postulate receives strong support from the observation that children with NF1 who do not exhibit plexiform neurofibromas upon first MRI examination are unlikely to develop new plexiform neurofibromas later in life (Nguyen et al. 2012). Hence, a high burden of internal neurofibromas may well be strongly associated with an increased MPNST risk and this should be taken into account when planning the clinical care of patients with *NF1* microdeletions.

### Increased risk of MPNSTs in patients with *NF1 *microdeletions

MPNSTs are rare soft tissue sarcomas occurring with an incidence of 0.001% in the overall (general) population (Ducatman et al. 1986) and are known to have an association with NF1. Thus, some 28–52% of patients with MPNSTs also have NF1 (Ducatman et al. 1986; Evans et al. 2002). The estimated lifetime risk of an MPNST in all NF1 patients is 8–13% (Evans et al. 2002, 2012) or 15.8% according to Uusitalo et al. (2016). However, individuals with *NF1* microdeletions have an even higher lifetime MPNST risk, in the range of 16–26% (De Raedt et al. 2003; Mautner et al. 2010). Further, MPNSTs may occur significantly earlier in patients with *NF1* microdeletions as compared with NF1 patients with intragenic mutations (De Raedt et al. 2003). These findings clearly indicate that patients with *NF1* microdeletions constitute a high-risk group for the development of MPNSTs. Hence patients with *NF1* microdeletions should be under regular surveillance from an early age.

MPNSTs are difficult to treat, particularly if they are at an advanced stage and have already metastasized. The complete surgical excision of non-metastatic MPNSTs represents the mainstay of an effective therapy but this is only possible if the tumour is detected at an early stage (reviewed by Karajannis and Ferner 2015). MPNSTs represent the biggest contributory factor to reduced life expectancy in NF1 (Evans et al. 2012). It may well be that an *NF1* microdeletion and high internal tumour load are independent risk factors for MPNST. Thus, patients with an *NF1* microdeletion and a high internal tumour load could represent an ultra-high risk group for MPNST. For this group of patients, long-term follow-up investigations using whole-body MRI and serial ^18^Fluorodeoxyglucose positron emission tomography (PET) scans are likely to be critically important to identify malignant transformation at an early stage (Salamon et al. 2015).

### Intellectual disability in patients with *NF1* microdeletions

An estimated, 4.8–8% of all NF1 patients are characterised by intellectual disability (mean full-scale IQ (FSIQ) <70), a somewhat higher proportion than the 2% observed in the normal population (Ferner et al. 1996; reviewed by North et al. 1997). Several studies have suggested that intellectual disability occurs disproportionately more frequently in *NF1* microdeletion patients than in the general NF1 population (Kayes et al. 1994; Rasmussen et al. 1998; Korf et al. 1999; Venturin et al. 2004b; reviewed by Mensink et al. 2006). However, some of the early studies were biassed by including mostly patients with a particularly severe phenotype and uncharacterised deletion size. Only in two studies, the frequency of intellectual disability has been analysed systematically by including exclusively non-mosaic patients with *NF1* microdeletions of the same size (type-1 deletions spanning 1.4-Mb) (Descheemaeker et al. 2004; Mautner et al. 2010). Intellectual disability was evident in eight of 21 patients (38%) with type-1 *NF1* deletions analysed by Mautner et al. (2010) and in two of 11 patients with type-1 *NF1* microdeletions (18%) investigated by Descheemaeker et al. (2004). Taken together, these findings indicate that intellectual disability is markedly more frequent in patients with type-1 *NF1* microdeletions as compared to patients with intragenic *NF1* mutations. Furthermore, borderline intellectual disability, characterised by a full-scale IQ (FSIQ) higher than 70 but lower than 85 (70 ≤ FSIQ ≤ 85), was noted in seven (33%) of the 21 type-1 *NF1* microdeletion patients analysed by Mautner et al. (2010) and in nine of the 11 type-1 *NF1* microdeletion patients (82%) investigated by Descheemaeker et al. (2004).

Patients with NF1, irrespective of their mutation type, have a mean FSIQ of 88-99 which is in the range of one standard deviation lower than the FSIQ of the general population (100 ± 15) (North et al. 1997; Krab et al. 2008). A mean FSIQ of 76.9 was documented in the 21 type-1 *NF1* microdeletion patients investigated by Mautner et al. (2010). This is similar to the mean FSIQ of 76.0 ascertained in the 11 type-1 *NF1* microdeletion patients analysed by Descheemaeker et al. (2004). By comparison, a mean FSIQ of 88.5 was determined in 106 NF1 patients without an *NF1* deletion (Descheemaeker et al. 2004). Despite the relatively small numbers of individuals available in these studies, the tentative conclusion to be drawn from them is that the mean FSIQ in patients with type-1 *NF1* microdeletions is markedly lower than the mean FSIQ in patients with intragenic *NF1* mutations. However, Descheemaeker et al. (2004) noted a considerable overlap regarding the range of the FSIQ observed in patients with *NF1* microdeletions (65–85) as compared with the range observed in patients without microdeletions (54–126), even though the average intelligence (as measured by FSIQ) of type-1 *NF1* microdeletion patients is generally lower than that of NF1 patients without large microdeletions.

### Overgrowth associated with *NF1* microdeletions

Stature is reduced to some extent in virtually all patients with intragenic *NF1* mutations. On average, adolescents and adults with NF1 are one standard deviation shorter than would be expected for their age and sex (Clementi et al. 1999; Szudek et al. 2000). Short stature, characterised by a height of more than two standard deviations below the predicted mean, is evident in 8–13% of children with NF1 (Szudek et al. 2000; Sbidian et al. 2012; Soucy et al. 2013) whilst a body height below the third percentile has been observed in 15% of children with NF1 (Clementi et al. 1999).

Short stature in children with NF1 and intragenic *NF1* mutations has been suggested to be caused by a paucity of growth hormone as a consequence of abnormal hypothalamic–pituitary axis function (Vassilopoulou-Sellin et al. 2000). Support for this hypothesis comes from the phenotype observed in conditional knockout mice with specific *Nf1* gene inactivation in neuroglial progenitor cells using the brain lipid-binding protein promoter (Hegedus et al. 2008). These mice exhibit significantly reduced body weight and anterior pituitary gland size caused by the loss of neurofibromin expression in the hypothalamus, leading to reduced production of growth hormone releasing hormone, pituitary growth hormone and liver-expressed insulin-like growth factor-1 (IGF1). Thus, it would appear that neurofibromin plays a critical role in hypothalamic–pituitary axis function and hence its loss may cause growth abnormalities in patients with NF1 (Hegedus et al. 2008).

In contrast to the reduced stature observed in most patients with intragenic *NF1* mutations, tall stature in adults and childhood overgrowth has been reported to occur frequently in patients with *NF1* microdeletions (van Asperen et al. 1998; Spiegel et al. 2005; Mensink et al. 2006; Pasmant et al. 2010). Tall-for-age stature, with height measurements at or above the 94th percentile, was noted in 46% of patients with germline type-1 *NF1* deletions (Mautner et al. 2010). Since intragenic *NF1* mutations lead to shorter height, and total loss of the *NF1* gene plus flanking genes often results in tall-for-age stature, it may be concluded that haploinsufficiency of a gene or genes co-deleted in patients with *NF1* microdeletions causes this overgrowth phenotype (Mautner et al. 2010). In a study of 21 patients with type-1 *NF1* microdeletions, overgrowth was most evident in preschool children (2–6 years, *n* = 10) (Spiegel et al. 2005). These findings were confirmed by Ning et al. (2016) who performed longitudinal growth measurements in 56 NF patients with *NF1* microdeletions and 226 NF1 patients with intragenic *NF1* mutations. Most height measurements in 2–18-year-old boys and girls with *NF1* microdeletions were greater than the median observed in non-deletion NF1 patients. However, extreme body height (more than three standard deviations above the mean) was still unusual among patients with *NF1* microdeletions (Ning et al. 2016). These authors also showed that children with *NF1* microdeletions were usually much taller than non-deletion NF1 patients after the age of 2 years. In early infancy, before the age of 2, the body length of microdeletion and non-deletion NF1 patients was found to be similar (Ning et al. 2016). The reasons for these differences in growth pattern are currently unknown.

### Dysmorphic facial features

Patients with *NF1* microdeletions frequently exhibit dysmorphic facial features not seen in patients with intragenic *NF1* mutations. These features include broad neck, hypertelorism, downslanted palpebral fissures, a broad nasal bridge and a coarse, sometimes fleshy facial appearance (Mensink et al. 2006 and references therein; Venturin et al. 2004b; Pasmant et al. 2010; Mautner et al. 2010). A dysmorphic facial appearance is the most commonly observed feature in patients with *NF1* microdeletions (Table 1) and is probably caused by haploinsufficiency for a gene or genes located within the deletion interval. The dysmorphic facial appearance as seen in patients with *NF1* microdeletions is generally absent in patients with intragenic *NF1* mutations. Using *Face2Gene* facial recognition software (http://www.fdna.com) may be helpful in characterising and quantifying the dysmorphic facial features observed in patients with *NF1* microdeletions, particularly if comparison is made with family members lacking the *NF1* microdeletion.

### Cardiovascular malformations

Several studies have reported the occurrence of heart defects in a proportion of patients with *NF1* microdeletions (Kayes et al. 1994; Tonsgard et al. 1997; Wu et al. 1997; Dorschner et al. 2000; Riva et al. 2000; Oktenli et al. 2003; Mensink et al. 2006; Venturin et al. 2004b; De Luca et al. 2007). However, the overall frequency of such defects in patients with *NF1* microdeletions has remained unclear. In the study of Mautner et al. (2010), eight (29%) of the 28 type-1 *NF1* deletion patients investigated had cardiovascular anomalies. The first detailed analysis of the prevalence of heart defects in patients harbouring *NF1* microdeletions was performed by Nguyen et al. (2013) who observed major cardiac abnormalities in 6 of 16 *NF1* microdeletion patients whereas none of 16 patients with intragenic *NF1* mutations exhibited heart defects. Consequently, it would appear that heart defects are significantly more common in patients with *NF1* microdeletions as compared to patients with intragenic *NF1* mutations. However, the type and frequency of the heart defects observed in patients with *NF1* microdeletions are quite heterogeneous, including pulmonic stenosis, ventricular septal defect, aortic stenosis, atrial septal defect, aortic stenosis, mitral valve prolapse or insufficiency, and hypertrophic cardiomyopathy (Table 2). Larger studies are clearly required to ascertain the type and the frequency of heart defects in patients with *NF1* microdeletions with more precision.

**Table 2:** Heart defects observed in patients with *NF1* microdeletions

Type of heart defect	Number of *NF1* microdeletion patients exhibiting the heart defect (reference)^a^
Pulmonic stenosis	1 (Tonsgard et al. 1997) 2 (Dorschner et al. 2000) 2 (Riva et al. 2000)
Ventricular septal defect	1 (Tonsgard et al. 1997) 1 (Venturin et al. 2004b) 1 (Nguyen et al. 2013)
Atrial septal defect	1 (Kayes et al. 1994) 1 (Dorschner et al. 2000)
Aortic stenosis	1 (Nguyen et al. 2013)
Aortic dissection	1 (Leppig et al. 1997)
Patent ductus arteriosus	1 (Upadhyaya et al. 1996)
Mitral valve prolapse	1 (Wu et al. 1997) 3 (Mensink et al. 2006) 1 (Venturin et al. 2004b) 1 (Oktenli et al. 2003)
Mitral valve insufficiency	1 (Venturin et al. 2004b) 2 (Nguyen et al. 2013)
Aortic valve insufficiency	1 (Nguyen et al. 2013) 1 (Dorschner et al. 2000)
Hypertrophic cardiomyopathy	1 (Mensink et al. 2006) 1 (Venturin et al. 2004b) 3 (Nguyen et al. 2013)
Intracardiac neurofibromas	2 (Nguyen et al. 2013)

^a^In the study of Nguyen et al. (2013), 6 of 16 *NF1* microdeletion patients had major cardiac abnormalities. Two of these patients exhibited several heart abnormalities: Patient 5a had an aortic and a mitral valve insufficiency as well as a cardiac tumour whereas patient 8a had aortic stenosis, mitral valve insufficiency and hypertrophic cardiomyopathy

## Co-deleted genes with the potential to influence the clinical phenotype in patients with *NF1* microdeletions

In addition to the deletion of the *NF1* gene, hemizygosity of any one of a number of genes located within the deletion interval at 17q11.2 may contribute to the clinical phenotype observed in patients with *NF1* microdeletions. Some of these genes may have tumour suppressive functions and their hemizygosity could predispose to an increased tumour risk or might facilitate tumour progression. There is certainly good evidence that biallelic *SUZ12* loss promotes MPNST progression (De Raedt et al. 2014; Lee et al. 2014; Zhang et al. 2014). Haploinsufficiency of other genes during early embryonic development and/or during later stages may, however, contribute to clinical sequelae such as overgrowth, reduced cognitive capabilities, heart defects and dysmorphic facial features. Thus, *RNF135* haploinsufficiency is associated with dysmorphic facial features and overgrowth (Douglas et al. 2007). The consequences of the deletion of the other genes located within the 1.4-Mb *NF1* microdeletion region (listed in Table 3) are less clear. This notwthstanding, the function, as well as the expression pattern of some of these genes, renders it highly likely that their loss impacts upon the *NF1* microdeletion-associated phenotype. The haploinsufficiency of some of these genes may even synergize with the loss of the *NF1* gene to aggravate the clinical manifestations of patients with *NF1* microdeletions. To estimate the likely consequence of haploinsufficiency of genes located within the *NF1* microdeletion region, the probability of loss-of-function (LoF) intolerance may be considered as calculated from the ExAC data set (Lek et al. 2016). The metric ‘probability of being LoF intolerant (pLI)’ separates genes into LoF intolerant (pLI ≥ 0.9) or LoF tolerant (pLI ≤ 0.1) categories. Importantly, *ATAD5*, *RAB11FIP4*, *LRRC37B* and *SUZ12* reside in the category of LoF intolerant genes suggesting that their haploinsufficiency in patients with *NF1* microdeletions is highly likely to have pathological consequences. By contrast, *TEFM*, *ADAP2*, *RNF135*, *EVI2A* and *UTP6* are LoF tolerant (Table 3). It cannot, however, be excluded that the hemizygosity of these latter genes may still contribute in one way or another to the *NF1* microdeletion phenotype.

**Table 3:** Protein-coding and microRNA genes located within the *NF1* microdeletion region at 17q11.2

Official HGNC gene symbol	Alternative names	MIM#	Official gene name	Probability of loss of function intolerance (pLI)
*CRLF3*	*FRWS; CRLM9; p48.2;CYTOR4; CREME*-*9*	614853	Cytokine receptor-like factor 3	0.98
*ATAD5*	*ELG1; FRAG1; C17orf41*	609534	ATPase family, AAA domain containing 5	1.00
*TEFM*	*C17orf42*	616422	Transcription elongation factor, mitochondrial	0.04
*ADAP2*	*CENTA2; Cent*-*b; HSA272195*	608635	ArfGAP with dual PH domains 2	0.00
*RNF135*	*L13; MMFD; REUL; Riplet*	611358	Ring finger protein 135p	0.00
*MIR4733*	None	—	microRNA 4733	
*NF1*	*WSS; NFNS; VRNF*	162200	neurofibromin 1	1.00
*OMG*	*OMGP*	164345	Oligodendrocyte myelin glycoprotein	0.86
*EVI2B*	*EVDB; CD361; D17S376*	158381	Ecotropic viral integration site 2B	0.18
*EVI2A*	*EVDA; EVI2; EVI*-*2A*	158380	Ecotropic viral integration site 2A	0.00
*RAB11FIP4*	*FIP4*-*Rab11; RAB11*-*FIP4*	611999	RAB11 family-interacting protein 4	0.99
*MIR193A*	*MIRN193; MIRN193A; mir*-*193a*	614,733	microRNA 193a	
*MIR365B*	*MIR365*-*2; mir*-*365b; MIRN365*-*2;hsa*-*mir*-*365b*	614,733	microRNA 365b	
*MIR4725*	*mir*-*4725*	—	microRNA 4725	
*COPRS*	*TTP1; COPR5; C17orf79; HSA272196*	616477	Coordinator of PRMT5 and differentiation stimulator	0.83
*UTP6*	*HCA66; C17orf40*	—	UTP6, small subunit processome component	0.00
*SUZ12*	*CHET9; JJAZ1*	613675	SUZ12 polycomb-repressive complex 2 subunit	1.00
*LRRC37B*	None	616558	Leucine-rich repeat containing 37B	0.95

^a^The ExAC browser (http://exac.broadinstitute.org/) provides the constraint metric termed “probability of loss of function” (pLI). To determine the pLI metric, the observed and expected variant counts for a given gene included in the ExAC dataset are considered. The closer the pLI value is to one, the more loss of function-intolerant the gene appears to be. A pLI value ≥ 0.9 is indicative of genes extremely intolerant of loss function variants

Our current knowledge of the genes located within the *NF1* microdeletion region that have the potential to modify the clinical phenotype in patients with *NF1* microdeletions, as concluded from previously published studies, is summarised below.

### *RNF135* may be involved in overgrowth and dysmorphic facial features

The *RNF135* gene located upstream of *NF1* represents a good candidate to account for the overgrowth phenotype observed in *NF1* microdeletion patients. This conclusion derives directly from the findings of Douglas et al. (2007), who analysed a cohort of 245 individuals with overgrowth, learning disability, dysmorphic facial features and detected *RNF135* mutations in 6 of them. The 245 patients investigated by Douglas et al. had been previously shown to be negative for *NSD1* mutations, a frequent cause of Sotos syndrome characterised by overgrowth, dysmorphic facial features and learning disability (Tatton-Brown et al. 2005). Five of the six *RNF135* mutation-positive patients identified by Douglas et al. harboured intragenic *RNF135* mutations whereas the other patient exhibited a microdeletion resulting from an NAHR event between NF1-REPa and NF1-REPb which included *RNF135* plus five other genes, but not *NF1* (Douglas et al. 2007). Inactivating mutations or entire gene deletions of *RNF135* do not appear to be frequent in patients with an overgrowth phenotype since *RNF135* mutations were not detected in another cohort of 160 *NSD1* mutation-negative patients with features of Sotos syndrome (Visser et al. 2009). Remarkably, among these 160 patients with suggested Sotos syndrome and overgrowth was a 4-year-old girl with dysmorphic facial features, two CALS and developmental delay who was found to have an *NF1* microdeletion. This finding indicates that an *NF1* microdeletion should be considered in the differential diagnosis of children with Sotos syndrome-associated features (Visser et al. 2009).

*RNF135* encodes an E3 ubiquitin ligase with an N-terminal RING finger domain and C-terminal SPRY and PRY motifs. It is expressed in many different tissues (Oshiumi et al. 2009). RNF135 ubiquitinates RIG-I (retinoic acid-inducible gene-I protein) and promotes its signal transduction capacity so as to produce antiviral type-I interferon 1 (Oshiumi et al. 2010). Owing to its ring finger domain and the PRY motif, RNF135 is likely to bind numerous proteins, suggestive of a wide range of functions. The mechanism underlying the overgrowth phenotype mediated by the loss of one *RNF135* copy is currently unclear and needs to be further investigated.

Importantly, patients with *RNF135* mutations exhibit dysmorphic facial features including hypertelorism, down-slanting palpebral fissures and a broad nasal tip giving rise to a facial appearance similar to that observed in patients with *NF1* microdeletions (Douglas et al. 2007). These findings suggest that *RNF135* haploinsufficiency may be responsible for the dysmorphic facial features observed in patients with *NF1* microdeletions. However, since only five patients with intragenic *RNF135* mutations but lacking *NF1* microdeletions have so far been reported, more extended genotype/phenotype studies are necessary to assess whether the dysmorphic facial features are indeed caused by *RNF135* mutations.

### *SUZ12* and its role in MPNST development in patients with *NF1* microdeletions

The increased risk of MPNSTs in patients with large *NF1* microdeletions is probably associated with hemizygosity of the *SUZ12* gene, located telomeric to *NF1* within the *NF1* microdeletion region (Fig. 1). *SUZ12* is frequently bi-allelically inactivated in MPNSTs suggestive of a tumour suppressor function in this tumour type (De Raedt et al. 2014; Lee et al. 2014; Zhang et al. 2014). As a component of the Polycomb repressive complex 2 (PRC2), the SUZ12 protein is involved in the epigenetic silencing of many different genes by establishing di- and tri-methylation of histone H3 lysine 27 (reviewed by Di Croce and Helin 2013). Loss of histone H3 lysine 27 trimethylation has been observed in 50–70% of MPNSTs. By contrast, H3 lysine 27 trimethylation is retained in benign neurofibromas and hence serves as a diagnostic marker for malignant transformation (Asano et al. 2017; Cleven et al. 2016; Prieto-Granada et al. 2016; Schaefer et al. 2016; Röhrich et al. 2016). The genes targeted by PRC2 regulate cell cycle progression, stem cell self-renewal, cell fate decisions and cellular identity. The expression changes of some of these genes consequent to the loss of PRC2 function appear likely to contribute to tumorigenesis (reviewed by Conway et al. 2015; Laugesen et al. 2016). Thus, PRC2 loss has been shown to amplify Ras-driven gene expression through epigenetic changes (De Raedt et al. 2014). Somatic inactivating mutations of *SUZ12* or other genes encoding PRC2 components have been detected in MPNSTs but not in benign neurofibromas and atypical neurofibromas. The latter are considered to be premalignant tumours with high potential to transform into MPNSTs (De Raedt et al. 2014; Lee et al. 2014; Zhang et al. 2014; Pemov et al. 2016). Consequently, loss of PRC2 function is important during malignant transformation and/or progression of MPNSTs. In patients with germline *NF1* microdeletions, one *SUZ12* allele is deleted in all cells and the probability of acquiring a somatic mutation of the remaining *SUZ12* allele is clearly going to be higher than acquiring two independent somatic *SUZ12* mutations (as would be necessary in NF1 patients with intragenic *NF1* mutations, or in patients without NF1 who exhibit sporadic MPNSTs). Hence, the constitutional deletion of one *SUZ12* allele represents a predisposing factor that contributes to the increased risk of MPNSTs in patients with *NF1* microdeletions.

### Other genes with known tumour suppressor function within the *NF1* microdeletion region

In addition to the *NF1* and *SUZ12* genes, patients with *NF1* microdeletions are also hemizygous for three other genes with putative tumour suppressor function: *ATAD5* and the microRNA genes *MIR193A* and *MIR365B*. Whilst the importance of *SUZ12* loss in MPNST progression has now been well documented, rather less is known about the co-deleted genes *ATAD5*, *MIR193A*, *MIR365B* and their involvement in MPNST pathogenesis. However, as deduced from their function, it is not unreasonable to suppose that haploinsufficiency of these genes could promote MPNST development in patients with *NF1* microdeletions as discussed below.

#### *ATAD5*

Another tumour suppressor gene located within the *NF1* microdeletion region is *ATAD5* (ATPase family AAA domain–containing protein 5) (Fig. 1). The ATAD5 protein is involved in the stabilisation of stalled DNA replication forks by regulating proliferating cell nuclear antigen (PCNA) ubiquitination during DNA damage bypass, thereby promoting the exchange of a low-fidelity translesion polymerase back to a high-fidelity replication polymerase (Lee et al. 2010, 2013). Mice haploinsufficient for Atad5 (Atad5^+/m^) display high levels of genomic instability (Bell et al. 2011). Embryonic fibroblasts from Atad5^+/m^ mice exhibit molecular defects in PCNA deubiquitination in response to DNA damage, as well as DNA damage hypersensitivity, high levels of genomic instability and aneuploidy. More than 90% of haploinsufficient Atad5^+/m^ mice developed tumours such as sarcomas, carcinomas and adenocarcinomas that exhibited high levels of genomic instability (Bell et al. 2011). Furthermore, somatic *ATAD5* mutations were identified in a subset of sporadic human endometrial tumours (Bell et al. 2011) as well as breast and ovarian tumour cell lines (Abaan et al. 2013). Hence, *ATAD5* is regarded as a tumour suppressor gene (Bell et al. 2011; Kubota et al. 2013). Rare germline missense variants of *ATAD5* with predicted pathogenicity have been reported to be enriched in patients with ovarian cancer as compared with controls (Maleva Kostovska et al. 2016). Targeted knockdown of *ATAD5* expression in human cell lines has been shown to confer sensitivity to DNA damaging agents and cause severe genomic instability (Sikdar et al. 2009). Consequently, we may surmise that ATAD5 functions as an important regulator of genome instability (Gazy et al. 2015). Taken together, *ATAD5* haploinsufficiency is likely to contribute to tumorigenesis in patients with *NF1* microdeletions, in particular MPNST pathogenesis, since these tumours exhibit a high degree of genome instability including numerous copy number variants as well as chromosomal rearrangements (Mantripragada et al. 2009; Beert et al. 2011 and references therein).

#### MicroRNA genes

MicroRNAs (miRNAs) can play a critical role during tumorigenesis by directly interacting with the 3′UTRs of specific target mRNAs and inhibiting their translation (reviewed by Lovat et al. 2011). MicroRNAs have also been implicated in NF1-associated tumorigenesis (Sedani et al. 2012). Four microRNA genes are located within the 1.4-Mb *NF1* microdeletion region, *MIR193A*, *MIR365B, MIR4725* and *MIR4733* (Fig. 1). One of these, *MIR193A,* encodes two mature miRNAs with well-known tumour suppressor functions; miR193a-3p and miR193a-5p are generated from the primary transcript by means of several maturation steps. The expression preference of miR193a-5p and miR193a-3p is likely to be determined by the Ago protein (reviewed by Tsai et al. 2016). According to the mirbase database (http://www.mirbase.org/), miR193a-3p is more abundantly expressed in human tissues than miR193a-5p. Several studies have indicated that miR193a-3p suppresses tumour development by silencing multiple target genes including *SRSF2*, *HIC2, HOXC9, PSEN1, LOXL4, ING5*, *KIT*, *PLAU* and *MCL1* (Tsai et al. 2016). miR193a-3p has been found to be down-regulated by hypermethylation in oral squamous cell carcinoma cell lines (Kozaki et al. 2008), non-small cell lung cancer (Heller et al. 2012; Wang et al. 2013; Liang et al. 2015; Ren et al. 2015), bladder cancer (Deng et al. 2014; Lv et al. 2014; Li et al. 2015), hepatocellular carcinoma (Salvi et al. 2013), *BRAF* mutation-positive malignant melanoma (Caramuta et al. 2010), acute myeloid leukaemia (Xing et al. 2015) and pleural mesothelioma (Williams et al. 2015). However, it is unclear whether the down-regulation of miR193a-3p is a cause or a consequence of tumorigenesis. Decreased expression of miR193a-3p has been found to be correlated with metastasis, apoptosis and proliferation in breast cancer cell lines (Iliopoulos et al. 2011; Tsai et al. 2016) and ovarian tumour tissue (Nakano et al. 2013). Moreover, miR193a-5p is known to possess tumour suppressor functions since it inhibits the growth of breast cancer cells (Tsai et al. 2016) and endometrioid endometrial carcinoma cells by down-regulation of the transcription factor YY1 (Yang et al. 2013). Both miR193a-5p and miR193a-3p suppress lung cancer cell migration and invasion by co-regulating the ERBB4/PIK3R3/mTOR/S6K2 signalling pathway (Yu et al. 2015). These findings indicate that miR193a-3p and miR193a-5p play a tumour suppressor role in many different tumour types. In particular, the putative tumour suppressor function of miR193a-3p in breast cancer cell lines is noteworthy since breast cancer occurs at an increased frequency in patients with NF1 (Sharif et al. 2007; Seminog and Goldacre 2013, 2015; Uusitalo et al. 2016). However, it is unknown whether the breast cancer risk is higher in patients with *NF1* microdeletions than in patients with intragenic *NF1* mutations.

The *MIR365B* gene, located within the *NF1* microdeletion region (Fig. 1), also encodes an miRNA with known tumour suppressor function, as evidenced by its ability to target specific transcription factors, such as NKX2-1 and TTF1, in non-small cell lung cancer (Qi et al. 2012; Kang et al. 2013; Sun et al. 2015). miR365 is down-regulated in colon cancer (Nie et al. 2012), cutaneous squamous cell carcinoma (Zhou et al. 2014, 2015), hepatocellular carcinoma (Chen et al. 2015), gastric cancer (Guo et al. 2013) and malignant melanoma (Bai et al. 2015a, b). By contrast, putative tumour suppressor functions of the other two miRNAs located within the *NF1* microdeletion region, encoded by *MIR4725* and *MIR4733,* respectively, have not so far been reported.

Taken together, the loss of the *MIR193A* and *MIR365B* genes in patients with *NF1* microdeletions may well contribute to tumorigenesis in these patients. However, miRNAs are not only involved in tumour development; they also play a role as key regulators of metabolic homeostasis and tissue differentiation (reviewed by Vienberg et al. 2017). Additional studies are necessary to investigate the influence of the hemizygous loss of the miRNA genes on the clinical phenotype associated with *NF1* microdeletions.

### Other genes that may contribute to tumour development in patients with *NF1* microdeletions

In addition to the deletion of *SUZ12* and *ATAD5*, hemizygosity for the genes *COPRS*, *UTP6* and *RNF135* may also contribute to the increased tumour risk associated with *NF1* microdeletions. Current knowledge about the function of these genes is summarised in the following paragraphs.

#### *COPRS*

*COPRS* is another gene located within the *NF1* microdeletion interval that may well play a role in MPNST development. *COPRS* encodes an adaptor protein that binds strongly to protein-arginine methyltransferase 5 (PRTM5) and to histone H4. By these means, COPRS recruits PRMT5 to chromatin and also modulates PRMT5 substrate specificity since PRMT5 bound to COPRS preferentially methylates histone H4 instead of histone H3 (Lacroix et al. 2008). COPRS binding to PRMT5 is essential for myogenic differentiation, possibly through altered targeting of PRMT5 to specific gene promoters (Paul et al. 2012). These observations suggest that the COPRS–PRMT5 complex regulates cell differentiation, a process that is frequently perturbed during tumorigenesis. MPNSTs often exhibit regions of divergent differentiation possibly including rhabdomyosarcomatous, chondral, glandular, neuroendocrine, gangliocytic and liposarcomatous components. These regions of divergent differentiation may be focal on a background of typical spindle-shaped tumour cells. For example, in some cases, rhabdomyosarcomatous differentiation may become predominant rendering even differential diagnosis very difficult (Guo et al. 2012). Aberrant regulation of the COPRS–PRMT5 complex due to COPRS haploinsufficiency may contribute to these divergent differentiation patterns. Increased expression of PRMT5 has been noted in a wide variety of cancer types (reviewed by Stopa et al. 2015), but further studies are necessary to investigate how *COPRS* haploinsufficiency in patients with *NF1* microdeletions could alter PRMT5 function, thereby contributing to tumorigenesis.

Analysis of the expression level of *COPRS* in MPNST cell lines has yielded inconsistent results both within and between studies (Bartelt-Kirbach et al. 2009; Pasmant et al. 2011). Overexpression of *COPRS* (five to 10-fold) was observed in two MPNST tissue samples from patients with intragenic *NF1* mutations as compared with cutaneous neurofibroma tissue (Bartelt-Kirbach et al. 2009). However, by contrast, these authors detected low expression of *COPRS* in an MPNST cell line, which was as low as the *COPRS* expression level in neurofibroma-derived fibroblast cell cultures. The MPNST cell line analysed by these authors was derived from an NF1 patient but the germline *NF1* mutation in this patient had not been determined. In similar vein, whilst Pasmant et al. (2011) observed high *COPRS* expression in a series of MPNST cell lines as compared with plexiform and cutaneous neurofibroma samples, in other MPNST cell lines, *COPRS* expression levels were as low as in plexiform and cutaneous neurofibromas (Pasmant et al. 2011). Unfortunately, these authors failed to specify the origin of the MPNST cell lines analysed, whether they were derived from NF1 patients and if so, whether they harboured *NF1* microdeletions. The inconsistent findings regarding the *COPRS* expression level in MPNSTs reported by Bartelt-Kirbach et al. (2009) and Pasmant et al. (2011) are difficult to interpret. It may be that MPNST cell lines are an inappropriate system in which to investigate *COPRS* expression, perhaps because these cell lines have been subject to massive in vitro selection and hence may not be representative of key stages of MPNST development in vivo. Thus, the analysis of *COPRS* expression should be performed using primary MPNST samples (with high proportions of tumour cells) from patients with *NF1* microdeletions to explore the role of *COPRS* during MPNST development or progression in *NF1* microdeletion patients.

#### *UTP6*

Tumorigenesis in patients with *NF1* microdeletions may also be influenced by haploinsufficiency of *UTP6,* located telomeric to *NF1* within the *NF1* microdeletion region (Fig. 1). The protein encoded by UTP6 is involved in apoptosome-dependent apoptosis and it has been suggested that UTP6 haploinsufficiency could render cells with *NF1* microdeletions less susceptible to apoptosis (Piddubnyak et al. 2007). UTP6 is required for ribosome synthesis (Bonnart et al. 2012) and is, as a component of the centrosome, required for centriole duplication and the establishment of a bipolar spindle ensuring proper chromosome segregation during mitosis (Fant et al. 2009; Ferraro et al. 2011). Somatic loss of the remaining *UTP6* allele in tumours of patients with *NF1* microdeletions may contribute to malignant transformation by increasing chromosome instability and aneuploidy.

#### *RNF135*

In addition to its influence on the childhood overgrowth phenotype and the dysmorphic facial features observed in patients with *NF1* microdeletions, *RNF135* haploinsufficiency may also promote tumorigenesis. The *RNF135* gene has been shown to be down-regulated in cells from malignant peripheral nerve sheath tumours (MPNSTs) and MPNST cell lines suggesting that *RNF135* loss is involved in conferring the increased MPNST risk characteristic of NF1 microdeletion patients (Pasmant et al. 2011). In glioblastomas, however, *RNF135* has been found to be upregulated and promotes the proliferation of human glioblastoma cells in vivo and in vitro via the ERK pathway (Liu et al. 2016). Furthermore, *RNF135* would appear to promote the expression of *PTEN* and *TP53* in tongue cancer SCC25 cells and *RNF135* overexpression inhibits the viability, proliferation, and invasion of these cells (Jin et al. 2016). Taken together, these findings suggest that changes in the dosage of *RNF135* might contribute to tumorigenesis although further studies are necessary to clarify this postulate.

### Haploinsufficiency of genes in *NF1* microdeletion patients and intellectual disability

#### *OMG*

Hemizygosity of a gene (or several genes) located within the *NF1* microdeletion region may contribute to the intellectual disability noted in patients with large *NF1* deletions (Venturin et al. 2006). A good candidate is the *OMG* gene which encodes the oligodendrocyte myelin glycoprotein (OMgp) involved in the regulation of synaptic plasticity (reviewed by Mironova and Giger 2013). Synaptic plasticity and structural changes of the synapse have been suggested to cause cognitive and functional defects observed in intellectual disability, autism spectrum disorders and schizophrenia (Bernardinelli et al. 2014).

OMgp is anchored to the myelin membrane through a glycosylphosphatidyl inositol lipid molecule and is expressed in neurons as well as oligodendrocytes (Habib et al. 1998; Raiker et al. 2010 and references therein). OMgp belongs to the group of myelin-associated inhibitor proteins (MAIPs) which act as central nervous system (CNS) regeneration inhibitors by preventing injured axons from regenerating beyond the injury site. Prototypical MAIPs, including OMgp, are expressed in the healthy as well as the injured brain and bind to the Nogo-66 receptor (NgR1) and the paired Ig-like receptor B (PirB) which appear to inhibit neurite outgrowth in the adult CNS (Atwal et al. 2008; Akbik et al. 2012; Geoffroy and Zheng 2014; Baldwin and Giger 2015). However, in the adult CNS, OMgp and other MAIPs also regulate neuronal morphology, dendritic spine shape and activity-driven synaptic plasticity by binding to their receptors as determined both by NgR1 and PirB knockout mouse models and human cell lines (McGee et al. 2005; Syken et al. 2006; Lee et al. 2008; Raiker et al. 2010; reviewed by Mironova and Giger 2013).

In addition to its function in the adult CNS, OMgp plays important roles during early stages of brain development before the onset of myelination, possibly by regulating neurogenesis (Martin et al. 2009). During normal mouse development, neuronal OMgp is expressed, from embryonic day E14 on, in growing axons during axonal tract formation following the maturation of cortical connexions (Gil et al. 2010). In primary hippocampal cultures of adult normal mice, OMgp is present in the neuronal membrane, synaptosomal fractions and axonal varicosities (Gil et al. 2010). OMgp-null mice show impaired myelination and thalamo-cortical projection (Gil et al. 2010) as well as hypomyelination of the spinal cord that correlates with lower propagation of ascending and descending electrical impulses (Lee et al. 2011). Even although OMgp-null mice may not represent a wholly appropriate model with which to ascertain the consequences of *OMG* haploinsufficiency in humans, data derived from this system are consistent with the view that OMgp plays a key role in axonal target specification and synaptic plasticity.

Many studies have indicated that dysfunction of synapse formation, shape or density and synaptic plasticity cause intellectual disability and neuropsychiatric disorders (reviewed by Pittenger 2013; Srivastava and Schwartz 2014). Since MAIPs and their receptors play important roles in regulating synapse formation and plasticity, altered expression or function of these proteins may contribute to intellectual disability and other brain disorders (Sinibaldi et al. 2004; Budel et al. 2008; Tews et al. 2013; Llorens et al. 2011; Willi and Schwab, 2013; Petrasek et al. 2014). Consequently, OMgp haploinsufficiency may well contribute to the intellectual disability observed in patients with *NF1* microdeletions. The negative effects of OMgp haploinsufficiency on synaptic plasticity could be additive in relation to the consequences of the loss of the *NF1* gene product neurofibromin, an important regulator of Ras signalling in the brain. At least 50% of the patients with intragenic *NF1* mutations suffer from intellectual disabilities manifesting as cognitive slowing, memory disturbances, difficulties in solving strategic problems, visuospatial impairment and deficits in motor coordination (Diggs-Andrews and Gutmann 2013; Violante et al. 2013). These symptoms are further aggravated in patients with *NF1* microdeletions who exhibit a significantly lower mean FSIQ than patients with intragenic *NF1* mutations (Descheemaeker et al. 2004).

Similar to the phenotype observed in patients with NF1, behavioural studies in Nf1-deficient mouse models indicated deficits in spatial learning and motor coordination (Shilyansky et al. 2010a, b; van der Vaart et al. 2011). These mouse models also revealed that increased Ras/MAPK (mitogen-activated protein kinases) signalling results in higher GABA (gamma-aminobutyric acid) release during learning causing deficits in hippocampal long-term potentiation (LTP) that could account for the spatial learning and memory deficits of these mutant mice (Costa et al. 2002; Cui et al. 2008). Hence, neurofibromin is an important Ras regulator in interneurons influencing hippocampal-dependent learning. Ras signalling in dendritic spines of pyramidal neurons is required for many forms of synaptic plasticity, including LTP, spine structural plasticity, and new spine formation (reviewed by Oliveira and Yasuda 2014). Consequently, *NF1* and *OMG* haploinsufficiency are likely to exert additive negative effects that are causally associated with the cognitive deficit evident in many patients with *NF1* microdeletions. Loss-of-function mutations in the *OMG* gene have not, however, been observed in patients with idiopathic intellectual disability (Venturin et al. 2006).

#### *RNF135*

*RNF135* haploinsufficiency may also contribute to the reduced cognitive capabilities observed in patients with *NF1* microdeletions. As mentioned earlier, *RNF135* encodes an E3 ubiquitin ligase; other ubiquitin ligase genes have already been implicated in the development of intellectual disability and autism (Tenorio et al. 2014; reviewed by Tastet et al. 2015).

A significantly increased frequency of genotypes carrying the rare allele of the *RNF135* missense variant rs111902263 (p.R115 K) has been observed in patients with autism as compared with healthy controls (*P* = 0.0019, odds ratio: 4.23, 95% confidence interval: 1.87–9.57) (Tastet et al. 2015). These authors also showed that the *RNF135* gene is expressed in the cerebral cortex of humans and mice. The *RNF135*-encoded protein, termed ‘Riplet’, regulates the cytosolic viral RNA receptors RIG-I by ubiquitination (Oshiumi et al. 2009, 2010, 2013). RIG-I and other RIG-I-like receptors contribute to innate antiviral immunity by inducing antiviral responses such as the production of type I interferons (IFNs) and proinflammatory cytokines (reviewed by Yoneyama et al. 2015). RIG-1 is upregulated in neurons upon viral infection and is an important component of the intrinsic antiviral pathways in the CNS (Nazmi et al. 2011). RIG-I knockdown in a mouse model was associated with reduced neural stem/progenitor cell proliferation (Mukherjee et al. 2015), suggesting that the RNF135 protein plays a role in neurogenesis. Further studies will be necessary to determine the role of RNF135/Riplet in neural stem/progenitor cells and during brain development, roles which may yet prove to be relevant in the context of *RNF135* haploinsufficiency and cognitive disability in patients with *NF1* microdeletions.

Intriguingly, the *ADAP2* gene, which is also located within the *NF1* microdeletion region, encodes another key regulator of RIG-I signalling. ADAP2, an ADP-ribosylation factor GTPase-activating protein (ArfGAP) with dual PH domains 2, plays an important role as a scaffold protein that couples different modules of RIG-I signalling, leading to the up-regulation of type-I interferon gene transcription in response to viral infection (Bist et al. 2016). The potential role of ADAP2 in the aetiology of cardiovascular malformations is discussed below.

### *ADAP2* and cardiovascular malformations in patients with *NF1* microdeletions

Hemizygosity of the *ADAP2* gene may contribute to the cardiovascular malformations observed in patients with *NF1* microdeletions. This conclusion is drawn from the observation that *ADAP2* is highly expressed during early stages of heart development in both mouse and human (Venturin et al. 2005, 2014). In zebrafish, *ADAP2* loss of function leads to circulatory deficiencies and heart shape defects or defective valvulogenesis (Venturin et al. 2014). The *ADAP*2-encoded protein acts as a GTPase-activating protein (GAP) of the ADP-ribosylation factor 6 (ARF6), a small GTPase involved in actin cytoskeleton remodelling. ADAP2 (centaurin-alpha2) is located in the cytoplasm but after EGF stimulation, it binds to the plasma membrane via phosphatidylinositols. Plasma membrane association of ADAP2 prevents ARF6 translocation to the plasma membrane. By these means, ADAP2 negatively regulates ARF6-mediated actin cytoskeleton reorganisation (Venkateswarlu et al. 2007). ADAP2 also interacts with beta-tubulin and stabilises microtubules (Zuccotti et al. 2012). As a microtubulin-associated protein expressed during early embryonic development in the central nervous system and in the heart, ADAP2 is likely to mediate microtubule cytoskeleton reorganisation during cell differentiation and migration. It is well known that the interaction between microtubules and the actin cytoskeleton in association with membrane-associated proteins regulates cell shape and cellular remodelling (reviewed by Basu and Chang 2007; Bezanilla et al. 2015). Since ADAP2 interacts with the microtubule/actin cytoskeleton, it may function as a cytoskeleton cross-talker that increases microtubule stability and modulates actin reorganisation and hence cellular morphology (Zucotti et al. 2012). Disturbances of the cytoskeletal organisation in myocytes during embryonal development may be responsible for the cardiovascular malformations observed in patients with *NF1* microdeletions. This postulate was reinforced by the findings of Venturin et al. (2014) who showed that in zebrafish, *ADAP2* is required for normal cardiac morphogenesis.

The protein product of the *NF1* gene, neurofibromin, is essential for embryonic cardiac valve formation and the study of mouse models has indicated that neurofibromin loss leads to cardiovascular lethality during early embryonic development; Nf1 regulation of Ras in the developing endothelium is required for regular development of endocardial cushions and the ventricular myocardium (Gitler et al. 2003; Ismat et al. 2006; Xu et al. 2009; Bajaj et al. 2012; Yzaguirre et al. 2015). Haploinsufficiency for both *NF1* and *ADAP2* may contribute either cooperatively or additively to the increased frequency of heart defects in patients with *NF1* microdeletions. Further, *SUZ12* and *UTP6* are highly expressed during the development of the human heart; it follows that their haploinsufficiency may also contribute to the increased prevalence of congenital heart defects in patients with *NF1* microdeletions (Venturin et al. 2005).

## Clinical phenotype in patients with *NF1* microdeletions: influence of mosaicism and deletion size

The presence of normal cells not harbouring an *NF1* microdeletion exerts a major influence on disease severity in patients with mosaic large *NF1* deletions. Depending upon the proportion of cells harbouring the deletion, the clinical phenotype can be very mild or may affect only certain body segments (Tinschert et al. 2000; Maertens et al. 2007). The frequency of somatic mosaicism is strongly associated with the type of *NF1* microdeletion. Type-2 *NF1* deletions, caused by NAHR between *SUZ12* and *SUZ12P1*, are frequently of postzygotic origin. Patients with postzygotic type-2 *NF1* deletions exhibit somatic mosaicism of cells with the deletion and normal cells not harbouring the deletion (Kehrer-Sawatzki et al. 2004; Steinmann et al. 2007; Vogt et al. 2012). It has been estimated that at least 63% of all type-2 *NF1* deletions are associated with somatic mosaicism (Vogt et al. 2012). Atypical *NF1* deletions are also frequently mosaic; among the 17 atypical *NF1* deletion patients investigated by Vogt et al. (2014), 10 patients (59%) exhibited somatic mosaicism with normal cells. By contrast, only a very low proportion (2–4%) of type-1 *NF1* microdeletions is associated with somatic mosaicism (Messiaen et al. 2011). Remarkably, patients with type-2 deletions exhibit tissue-specific differences in the proportion of cells with the deletion (termed del^(+/−)^ cells), whereas the proportion of del^(+/−)^ cells is very high (94–99%) in the blood of these patients, and much lower proportions of del^(+/−)^ cells are evident in urine samples (24–82%) (Roehl et al. 2012). Since mosaic type-2 *NF1* microdeletions occur in most instances during early embryonic development, the tissue-specific differences in the proportion of del^(+/−)^ cells should result from cell type-specific selection.

Genotype–phenotype correlations in patients with mosaic *NF1* microdeletions are difficult to perform because the variable proportion of normal cells in different tissues is likely to influence the expression of clinical symptoms. The proportion of normal cells is difficult to assess and may vary from tissue to tissue and from patient to patient. Unfortunately, only a small number of patients with mosaic *NF1* deletions have been analysed in any detail. None of the eight patients with mosaic type-2 *NF1* microdeletions exhibited facial dysmorphism, nor was there any evidence of delayed cognitive development and/or learning disabilities, cognitive impairment, congenital heart disease, hyperflexibility of joints, large hands and feet, muscular hypotonia or bone cysts, all of which are frequently observed in patients with germline type-1 *NF1* microdeletions (Table 1). Furthermore, externally visible and internal plexiform neurofibromas were significantly less prevalent in patients with mosaic type-2 *NF1* microdeletions as compared with patients carrying constitutional (germline) type-1 *NF1* microdeletions (Kehrer-Sawatzki et al. 2012). These differences in clinical phenotype are unlikely to be caused by the differing extent of type-1 and type-2 deletions. Even although only two patients with non-mosaic type-2 *NF1* microdeletions have so far been analysed in terms of their clinical phenotype (Vogt et al. 2011), it may be concluded that patients with non-mosaic type-2 deletions exhibit most of the clinical features that have been reported in individuals with germline type-1 *NF1* deletions. Thus, a severe disease manifestation is not confined to patients with type-1 *NF1* deletions but may also occur in individuals with non-mosaic type-2 *NF1* deletions. The loss of the *LRRC37B* gene, associated with type-1 microdeletions but not with type-2 microdeletions, is unlikely to exert a major influence on the clinical phenotype. We conclude that the less severe clinical phenotype observed in patients with mosaic type-2 *NF1* microdeletions is unrelated to the extent of the deletion but is instead associated with the presence of normal cells that lack the microdeletion. Nevertheless, an increased risk of MPNSTs may also exist for patients with mosaic type-2 *NF1* microdeletions and plexiform neurofibromas, since most MPNSTs develop from pre-existing plexiform neurofibromas (Tucker et al. 2005) and the concomitant loss of *NF1* and *SUZ12* in plexiform neurofibroma cells harbouring the type-2 *NF1* microdeletion increases the likelihood of malignant transformation.

The extent of the deletion may nevertheless be important in the context of genotype-phenotype relationships in patients with *NF1* microdeletions. It has been noted that patients with very large atypical *NF1* deletions that encompass several Mb, much larger than the classical 1.4-Mb spanning type-1 *NF1* deletions, exhibit very severe disease manifestations associated with many additional clinical features that are not generally found to be associated with type-1 *NF1* deletions (Upadhyaya et al. 1996; Cnossen et al. 1997; Dorschner et al. 2000; Kehrer-Sawatzki et al. 2003; Pasmant et al. 2008). However, these deletions were very heterogeneous in size and hence genotype–phenotype analyses are scarcely feasible. More interesting in this regard are shorter deletions with recurrent breakpoints such as type-3 *NF1* microdeletions. These deletions encompass only 1-Mb and do not include the five functional genes (*CRLF3*, *ATAD5, TEFM, ADAP2* and *RNF135*) located centromeric to NF1-REPb (Fig. 1). However, only eight type-3 *NF1* deletions have so far been identified by means of high-resolution breakpoint analysis (Bengesser et al. 2010; Pasmant et al. 2010; Messiaen et al. 2011). Unfortunately, the clinical data from these eight patients are far from comprehensive or completely unavailable. Intellectual disability or cognitive impairment was observed in four of these eight patients with type-3 *NF1* microdeletions. Consequently, a gene (or genes) influencing the cognitive capabilities in these patients is located either within the *NF1* gene itself (e.g. *OMG*) or located telomeric to *NF1*; *OMG* is probably the best candidate for such an influence, by virtue of its function. Remarkably, dysmorphic facial features were observed in six patients from whom clinical phenotypic data were available (Bengesser et al. 2010; Pasmant et al. 2010). Since the *RNF135* gene was not deleted in these patients, it would appear that *RNF135* haploinsufficiency cannot be held responsible for the dysmorphic facial features in these individuals. The *RNF135* gene is located 46-kb upstream of the centromeric breakpoint of type-3 *NF1* deletions. However, it cannot be unequivocally excluded that a regulatory element which influences *RNF135* expression has been deleted in patients with type-3 *NF1* deletions. The deletion of such a regulatory element could have impaired *RNF135* expression in those patients with type-3 deletions, a postulate which remains to be investigated. Unfortunately, since it is not yet known if patients with type-3 *NF1* deletions are affected by childhood overgrowth or tall stature as adults, no further conclusions can be drawn concerning *RN135* haploinsufficieny and its role in height determination in patients with *NF1* deletions. Detailed clinical characterisation of a larger number of patients with type-3 *NF1* deletions would be necessary to assess the contribution of the genes listed in Table 1 to the clinical phenotype associated with large *NF1* deletions.

## The clinical phenotype in *NF1* microdeletion vs. *NF1* microduplication patients

For many disease-associated microdeletions encompassing several hundred kb, the reciprocal microduplications have been identified. In most instances, microdeletions and the reciprocal microduplications differ in terms of the associated clinical phenotype (reviewed by Vissers and Stankiewicz 2012; Weise et al. 2012). Microduplications reciprocal to *NF1* microdeletions are not associated with a classical NF1 phenotype but instead with developmental delay and learning disabilities as the major clinical features. So far, 29 *NF1* microduplication carriers have been reported, 18 of them were unrelated cases (Lu et al. 2007; Grisart et al. 2008; Moles et al. 2012; Coe et al. 2014; Kehrer-Sawatzki et al. 2014). None of the individuals with an *NF1* microduplication so far reported exhibited neurofibromas or other NF1-associated tumours. Only two of the 29 *NF1* microduplication carriers had CALS. However, these CALS were atypical, with irregular borders and nonhomogeneous pigmentation which is not generally characteristic of those CALS typically seen in patients with NF1 (Kehrer-Sawatzki et al. 2014). One of the two *NF1* microduplication patients with CALS fulfilled the diagnostic criteria for NF1 because he not only had ten CALS but also Lisch nodules. Since only blood cells from this patients were available for investigation, it could not be excluded that the *NF1* microduplication observed in this patient was of postzygotic origin and that the patient might also harbour cells in his body that contained the reciprocal *NF1* microdeletion (rather than the *NF1* microduplication). The potential co-occurrence of cells with the reciprocal *NF1* microdeletion could have been responsible for the CALS and Lisch nodules observed in this patient. Furthermore, it could not be excluded that somatically acquired *NF1* mutations in melanocyte progenitor cells contributed to the occurrence of CALS and Lisch nodules in this individual. Since melanocytes from CALS of this patient could not be investigated, his clinical manifestations are difficult to interpret with regard to the underlying mutation (Kehrer-Sawatzki et al. 2014). Nevertheless, the analysis of the other 28 *NF1* microduplication carriers reported to date implies that these duplications do not cause a classical NF1 phenotype.

Importantly, the clinical phenotype associated with *NF1* microdeletions is fully penetrant; clinically unaffected individuals with germline *NF1* microdeletions have not been reported. By contrast, three carriers of familial *NF1* microduplications have been observed who, according to the authors, do not exhibit any obvious clinical signs (Grisart et al. 2008; Moles et al. 2012). However, *NF1* microduplications are very unlikely to be frequent neutral copy number variants since they have not been observed in a total of 30,134 control individuals (Shaikh et al. 2009; Cooper et al. 2011; Moles et al. 2012; Coe et al. 2014). In a disease context, *NF1* microduplications have been observed in 14 unrelated individuals identified from among a total of 77,902 patients who were investigated by array CGH due to developmental delay (Moles et al. 2012; Coe et al. 2014).

*NF1* microdeletions are estimated to occur with a frequency of approximately 1 in 60,000 individuals, calculated on the basis that large *NF1* deletions are observed in ~5% of all patients with NF1 which occurs with an incidence of ~1 in 3000. *NF1* microduplications were not observed in 30,134 control individuals analysed by array CGH (Shaikh et al. 2009; Cooper et al. 2011; Moles et al. 2012; Coe et al. 2014), but this number of individuals is still too low to estimate the frequency of *NF1* microduplications in the general population or to assess whether *NF1* microdeletions are more frequent than *NF1* microduplications.

## Conclusions

*NF1* microdeletions are often associated with a severe clinical phenotype characterised by features not observed at all (or at significantly lower frequency) in patients with intragenic *NF1* mutations. Although many published studies have described the *NF1* microdeletion-associated phenotype, what is lacking are large studies comparing NF1 patients with and without *NF1* microdeletions according to standardised evaluation criteria to ensure that the same analytical methods are identically applied in the investigation of both patient groups. Ideally, such studies should be performed by comparing patients with *NF1* microdeletions and patients with intragenic loss-of-function *NF1* mutations to minimise the effects of mutation severity. Further, these studies should include sufficiently high numbers of patients to perform meaningful statistical analysis whilst the age of the patients should be matched since many NF1-associated features are age-related. This would be best performed as a multicenter collaborative study to collect large numbers of patients with different *NF1* microdeletion types to analyse the different deletion types separately. Comprehensive comparative analyses of this kind could help to answer several open questions that have not so far been systematically addressed, e.g., whether an early (pre-pubertal) onset of growth of multiple cutaneous neurofibromas is significantly more prevalent in children with *NF1* microdeletions as compared to children with intragenic *NF1* mutations. In addition, a comparative analysis including a large number of age-matched adult patients is urgently required to ascertain whether high numbers of cutaneous neurofibromas (*N* > 1000) occur significantly more often in patients with *NF1* microdeletions than in patients with intragenic mutations. Clearly, these and other analyses of the genotype–phenotype relationship should be performed so as to include patients with non-mosaic *NF1* microdeletions with well characterised deletion breakpoints to assess the number and identity of the co-deleted genes. The most frequently occurring type-1 *NF1* deletions are important with regard to extended genotype-phenotype correlations since they are the easiest group in which to discern such correlations; however, the less frequent but recurrent type-3 *NF1* deletions are also of interest. Analyses of a larger number of patients with type-3 *NF1* microdeletions would be necessary to determine the influence of genes such as *RNF135* on the overgrowth and dysmorphic facial features, or the influence on the deletion-associated phenotype of other genes not rendered hemizygous by the type-3 *NF1* deletions (Fig. 1). Although the deletion of *SUZ12* may well predispose patients with *NF1* microdeletions to malignancy, in particular to the development of MPNSTs, the reasons for the disproportionately higher frequency of benign plexiform, subcutaneous and spinal neurofibromas in patients with *NF1* microdeletions is still unclear. It is possible that deleted genes other than *NF1* may also promote tumorigenesis and such additive effects could be investigated by the targeted knockout of individual genes located within the *NF1* microdeletion interval using the CRISPR/Cas9 system in human cells. Similar experiments might also be performed with mouse cells but it should be appreciated that the genomic region on mouse chromosome 11 orthologous to the *NF1* microdeletion region exhibits differences in both the number and arrangement of genes as compared with the human genomic region at 17q11.2 (Jenne et al. 2003).

Although *NF1* microdeletion patients as a group exhibit a more severe clinical phenotype than that generally exhibited by patients with *NF1* intragenic lesions, a certain degree of variability in terms of the expression of clinical symptoms is observed when individual patients with *NF1* microdeletions are compared, even in cases where their germline deletions are identical. In patients with intragenic *NF1* mutations, the level of expression of the wild-type *NF1* allele has been suggested to impact upon the clinical phenotype since skewed allele-specific expression of the *NF1* gene has been observed in healthy individuals (Hoffmeyer et al. 1995; Cowley et al. 1998; Jentarra et al. 2012). It would be important to investigate whether the phenotypic variability observed between patients with *NF1* microdeletions might also be caused by differences in expression of the wild-type alleles of the genes that are present in only one copy owing to the large *NF1* deletion. Such analyses, as well as an extended comparative analysis of the clinical phenotype of NF1 patients with and without *NF1* microdeletions, would be necessary to improve our understanding of the mechanisms involved as well as to characterise the deletion-associated phenotype in a more systematic and comprehensive manner.
